# Optimizing interactions to protein binding sites by integrating docking-scoring strategies into generative AI methods

**DOI:** 10.3389/fchem.2022.1012507

**Published:** 2022-10-19

**Authors:** Susanne Sauer, Hans Matter, Gerhard Hessler, Christoph Grebner

**Affiliations:** Synthetic Molecular Design, Integrated Drug Discovery, Sanofi, Frankfurt, Germany

**Keywords:** artificial intelligence, drug design, structure-based design, docking, scoring functions, machine learning

## Abstract

The identification and optimization of promising lead molecules is essential for drug discovery. Recently, artificial intelligence (AI) based generative methods provided complementary approaches for generating molecules under specific design constraints of relevance in drug design. The goal of our study is to incorporate protein 3D information directly into generative design by flexible docking plus an adapted protein-ligand scoring function, thereby moving towards automated structure-based design. First, the protein-ligand scoring function RFXscore integrating individual scoring terms, ligand descriptors, and combined terms was derived using the PDBbind database and internal data. Next, design results for different workflows are compared to solely ligand-based reward schemes. Our newly proposed, optimal workflow for structure-based generative design is shown to produce promising results, especially for those exploration scenarios, where diverse structures fitting to a protein binding site are requested. Best results are obtained using docking followed by RFXscore, while, depending on the exact application scenario, it was also found useful to combine this approach with other metrics that bias structure generation into “drug-like” chemical space, such as target-activity machine learning models, respectively.

## Graphical Abstract

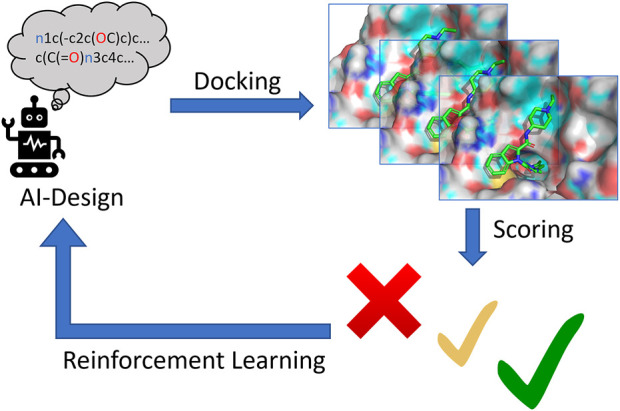

## 1 Introduction

Finding and optimizing promising lead molecules with high affinity for a particular protein is an important prerequisite for successful drug discovery. In addition to high throughput screening, virtual screening (Sotriffer et al., 2011; Stumpfe and Bajorath, 2020; Walters and Wang, 2020) is critical for identification of such compounds. In virtual screening, advanced computational strategies are applied to search collections of existing or virtual molecules (Muegge and Oloff, 2006; Hoffmann and Gastreich, 2019; van Hilten et al., 2019; Walters, 2019; Grebner et al., 2020a). Here, collections of virtual compounds are typically built from well-established chemical reactions and available building blocks to increase the likelihood of potential synthesis (Lyu et al., 2019). Hence, often synthetic success rates >80% are reported in the literature (Hoffmann and Gastreich, 2019; Lyu et al., 2019; van Hilten et al., 2019; Walters, 2019).

Likewise, *de novo* design (Schneider and Schneider, 2016; Schneider and Clark, 2019) also serves to sample the vast chemical space for active molecules. As the identification of chemical motifs is often not restricted by availability of building blocks or pre-defined chemical reactions, designed molecules are often challenging to synthesize (Hartenfeller et al., 2012; Gao and Coley, 2020).

Any automated design for compound structures with good affinity against a biological target of interest consists of two tasks, structure generation and scoring of the generated structures with a reward function to select candidates for synthesis. Dedicated software programs have been developed towards automation of some parts of this workflow including different fragment linking and growing strategies (Böhm, 1992; Gillet et al., 1993; Stahl et al., 2002; Schneider and Fechner, 2005; Dean et al., 2006). This led to a variety of *de novo* design approaches (Böhm, 1992; Gillet et al., 1993; Stahl et al., 2002; Schneider and Fechner, 2005; Dean et al., 2006; Todorov et al., 2006; Mauser and Guba, 2008; Hartenfeller et al., 2011).

In recent years, artificial intelligence (AI) based generative methods employing in particular neural networks provided a novel view on the creation of chemical structures under defined constraints. Several of these models are already applied in drug design settings (Chen et al., 2018; Hessler and Baringhaus, 2018; Grebner et al., 2020b). These include sampling of novel structures using recurrent neural networks (RNN) (Olivecrona et al., 2017; Popova et al., 2018; Arús-Pous et al., 2019; Brown et al., 2019), re-training of RNNs with collections of virtual structures (libraries) using transfer learning (Segler et al., 2018), using generative adversarial networks (GANs) (Sanchez-Lengeling et al., 2017; Guimaraes et al., 2018; Prykhodko et al., 2019) or reinforcement learning (RL) (Olivecrona et al., 2017; Popova et al., 2018; Segler et al., 2018; Ståhl et al., 2019), and autoencoders (Blaschke et al., 2018; Gómez-Bombarelli et al., 2018; Jin et al., 2019). Moreover, fragment-based reinforcement learning approaches based on an actor–critic model for generating structures have also been developed (Ståhl et al., 2019). Typically, the actor and the critic are both modeled with bidirectional long short-term memory (LSTM) networks (Ståhl et al., 2019).

Recurrent neural networks (RNN) (Goodfellow et al., 2016), originally applied for natural language processing, can process any sequential input like SMILES strings (Weininger, 1988) as “chemical language.” Typically, an initial model is trained with large chemical databases in SMILES representation as references. SMILES strings and characters are treated in analogy to “words”. RNN can learn the distribution of individual characters from the reference set. For sampling, the RNN is then initialized with a random token and each following character is computed by a multinomial sampling of the probability distribution in the model. This produces variability of the sampled structures. Once an end token is detected, the SMILES string is completed, with the complete SMILES string representing the generated structure. However, there are some disadvantages to this simple approach due to the complex grammar and lack of chemistry knowledge in SMILES. First, a large amount of reference data is needed to learn the generation of valid SMILES strings. Then, chemical motifs such as scaffolds and functional groups are not represented, and a chemical structure can be denoted by many different SMILES. Despite these issues, the SMILES-RNN approach has already been successful in design applications. Some disadvantages are accounted for by using molecular graphs (Jin et al., 2019) or fragments (Ståhl et al., 2019) as alternative molecular representations. Furthermore, different ways to encode chemical structures were recently developed, e.g., an improved SMILES-like description named DeepSmiles (O'Boyle and Dalke, 2018) or a method called SELFIES (Krenn et al., 2020).

Acceptable molecule structures in *de novo* design must fit to a desired property profile with high affinity to the desired target and favorable ADMET and physicochemical properties. These properties can be learned either indirectly from related molecules using transfer learning techniques (Amabilino et al., 2020) or directly from a scoring function, which computes a score for a given molecule, as implemented in reinforcement learning and particle swarm optimization (Olivecrona et al., 2017; Popova et al., 2018; Segler et al., 2018; Jin et al., 2019; Ståhl et al., 2019). In the second case, compounds are assessed using these properties to guide the design process. Machine learning models are here a natural choice, as they capture complex molecular properties in a model derived from the chemical structures of the ligands (Chen et al., 2018; Merk et al., 2018; Popova et al., 2018; Schneider, 2018; Wenzel et al., 2019; Zhavoronkov et al., 2019). Their potential difficulty is that a large set of ligands and affinities must be known, before a predictive model can be derived. Especially for novel target proteins, this is not always the case, while for ADMET properties, many validated models have already been described (Wenzel et al., 2019; Goller et al., 2020; Aleksić et al., 2021; Grebner et al., 2021). The design guided by those models often only explores the already known chemical space for that particular target. Alternative approaches have explored 3D shape similarity (Grant et al., 1996; Rush et al., 2005) to guide the design process (Grebner et al., 2020b; Papadopoulos et al., 2021).

Fewer examples have been reported, in which a protein 3D structure is directly employed for AI-based design. One obvious strategy relies on molecular docking approaches. For example, Dockstream has been added to REINVENT2.0 as a structure-based design component (Guo et al., 2021) with the goal to retain key protein-ligand interactions, to discard those design results with clashes to the binding site, and to explore additional subpockets for better overall performance in the scaffold-hopping scenario. Docking as reward for AI design was also used in the sample-and-dock pipeline (Xu et al., 2021a) that interfaces the junction-tree-variational autoencoders (Jin et al., 2019) with the docking engine rDock (Ruiz-Carmona et al., 2014). The DOCKSTRING bundle provides a benchmark how different machine learning algorithms, including de-novo-design methods, perform with molecular docking (García-Ortegón et al., 2022). Furthermore, the program OptiMol for optimization of binding affinities also integrates a docking evaluation in combination with SELFIES autoencoders (Boitreaud et al., 2020). In addition, the deep learning-based molecular generator, SBMolGen was reported to integrate a recurrent neural network, a Monte Carlo tree search, and docking simulations (Ma et al., 2021). A different structure-based *de novo* design strategy using 3D deep generative models was also recently described with the program DeepLigBuilder (Li et al., 2021). Here, ligand structures are directly generated within the binding site and scored using refinement docking of this initial molecule, which appears to be much faster than standard docking. Moreover, a prediction model for docking scores from SMILES as reward function for molecular design was implemented in the program V-dock (Choi and LeeV-Dock, 2021). Finally, in a retrospective design study for the GPCR DDR2, Glide and its Glide-SP score were directly integrated into the REINVENT generative approach (Thomas et al., 2021). Some other approaches directly use a geometrical representation of the protein binding pocket without explicit docking for generative AI (Skalic et al., 2019; Xu et al., 2021b).

The goal of our present study is to incorporate a state-of-the-art docking engine plus a protein-ligand adapted scoring function into our AI-based *de novo* design workflow to enable structure-based lead optimization. To this end, we have selected Glide-XP (Friesner et al., 2004; Halgren et al., 2004; Friesner et al., 2006) as one of the industry standards for structure-based design. Glide-XP differs from Glide-SP in its high accuracy with respect to pose prediction and affinity prediction (Friesner et al., 2006). Different post-processing schemes to obtain a reward term from docking were assessed for their usefulness in generative AI. In addition to the Glide-XP scoring term (Friesner et al., 2006) (gscore), a size-corrected term was used (ligand_efficiency_sa). Furthermore, we have developed a protein-ligand scoring function (RFXscore) based on individual Glide and Glide-XP scoring terms, RDKit ligand descriptors, and cross-terms of both. The “refined set” of the PDBbind 2019 database with more than 4,000 protein-ligand complexes and affinities served for training and validation. This RFXscore helps to improve ranking of active ligands and to discriminate between actives and inactives in comparison to the pure Glide-XP score.

We then study the impact of these reward methods alone or in combination with additional terms (e.g., target protein machine learning models, QED scores) as drivers of reinforcement learning on quality and chemical diversity of the newly generated molecules. In a retrospective exercise from a typical structure-based design project, we analyze to which extent molecules for project advancement can be automatically generated, which are drug-like and cover new chemical space. Hence, an improved picture of the usefulness of incorporating 3D protein information combined with a high-quality protein-ligand scoring function as reward term into generative AI design approaches emerges.

## 2 Materials and methods

### 2.1 Generative methods

Two generative engines have been used for compound generation, namely REINVENT and Lib-INVENT. REINVENT creates new molecules from scratch and requires several hundred steps to reach convergence, even with simple reward functions like 2D-fingerprint-based similarity. As 3D docking of batches of several hundred molecules per reinforcement learning (RL) iteration is a very time-consuming process, we first focused our evaluations on Lib-INVENT as structure generation engine. Therefore, the comparison of different structure-based scoring functions was performed with Lib-INVENT, which samples molecules from a given scaffold. As the possible chemical space is smaller, convergence is reached much faster. Since the architectures of REINVENT and Lib-INVENT are similar, conclusions obtained from Lib-INVENT might be transferable to REINVENT. This is demonstrated in final generation runs with the best scoring scheme in combination with REINVENT.

#### 2.1.1 Lib-INVENT

**FIGURE 1:**
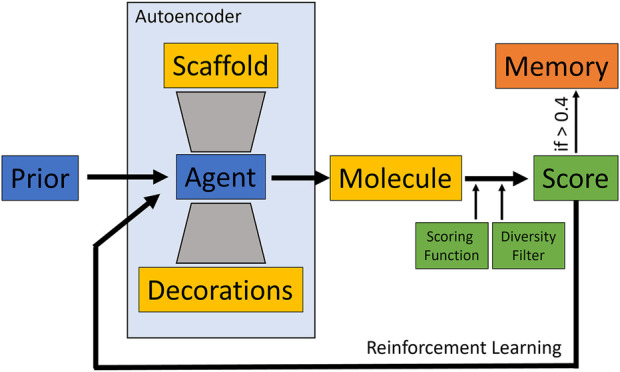
Overview of the Lib-INVENT training workflow. The agent takes a molecular scaffold as input and samples some decorations. Scaffold and decorations are then combined to form a molecule that is scored by the user-defined scoring function. This score is then scaled down according to the desired diversity filter to obtain the final score which is used to improve the model. If the score is above 0.4, the molecule is stored in memory (Fialkova et al., 2021).

To evaluate the behavior of the different scoring functions, the Lib-INVENT framework was used as a structure generator (Fialkova et al., 2021). Lib-INVENT is a modification of REINVENT for scaffold decoration. It contains two neural networks of the same architecture, prior and agent. The prior is trained only once and provides general chemistry knowledge, while the agent is driven towards a specific task that is defined by the scoring function. In case of Lib-INVENT, both networks are autoencoders that take a predefined molecular scaffold as input. This network will then generate decorations which are added to the scaffold at specified attachment vectors to form a complete chemical structure matching the predefined score. Both input scaffold and output decorations are encoded as SMILES strings (Fialkova et al., 2021). For an overview of the model training workflow see Figure 1.

**FIGURE 2:**
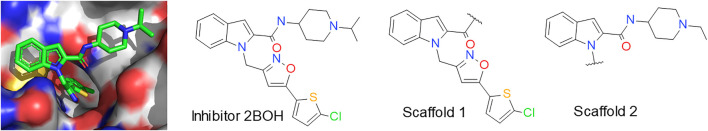
Inhibitor from PDB entry 2BOH (Nazaré et al., 2005a). The left image shows the 3D structure of the inhibitor in the pocket illustrated in PyMol (The PyMOL, 2022), on the right the two scaffolds used for Lib-INVENT are displayed. The atoms in the surface are colored by their element types.

As prior network, we used the reaction-based model provided in the Lib-INVENT repository (Fialkova and Patronov, 2022). It has been pre-trained on a cleaned subset of the ChEMBL database, sliced according to chemical reactions (Fialkova et al., 2021). The agent is initialized as a copy of the prior. The score is computed by the different scoring functions described below and a diversity filter that evaluates the diversity of the generated compounds as follows: Every generated molecule with a score above 0.4 is stored in memory. If a molecule is already in memory, the score from the scoring function is scaled down by a factor of 0.5 (Fialkova et al., 2021). This way, repeated generation of the same compound is punished by lower scores. This drives the process towards previously unexplored regions of chemical space, resulting in a higher diversity of generated compounds. Training was run for 100 epochs with a learning rate of 0.0001 and a batch size of 128. Two input scaffolds were given. They were created by cutting off the two sidechains of the fXa inhibitor from structure 2BOH of the Protein Data Bank (see Figure 2). (Nazaré et al., 2005a; Nazaré et al., 2005b) In scaffold 2, the isopropyl group was converted to ethyl by removing one carbon atom in order to lower the molecular weight while maintaining key interactions.

#### 2.1.2 REINVENT

REINVENT is a *de novo* generation method for molecular structures based on recurrent neural networks (RNNs) that employ SMILES strings (Weininger, 1988) as input and output (Olivecrona et al., 2017). The first RNN, called prior, is trained on a large number of molecules in order to learn general rules reflecting desirable chemistry. After training of this prior, RL is applied to narrow the chemical space of the generated structures. For this purpose, a second RNN, called agent, is initialized as a copy of the prior. Furthermore, a scoring function (also termed “reward function”) is introduced that modifies the output probabilities of the agent in a way that high scoring molecules have a higher probability to be sampled (Olivecrona et al., 2017). For an overview of the training workflow see Supplementary Figure S1.

It should be noted that the term “scoring” herein refers to reward functions guiding generative methods, unless noted otherwise. This should help to differentiate from “classical” protein-ligand scoring functions to guide docking engines (Sotriffer and Matter, 2011).

In this work, we used a prior network trained on the combined set of ChEMBL24, Enamine REAL space and the Sanofi compound collection as described previously (Grebner et al., 2020b). We used the most promising scoring functions from the Lib-INVENT runs to start REINVENT computations with 1,600 epochs.

In order to speed up convergence, we also used transfer learning to pre-train the prior network. For this, the ChEMBL database was searched using FastROCS (Openeye, 2022) with 2BOH and four other co-crystallized drugs for coagulation factor Xa (fXa) as queries. 464 molecules with a TanimotoCombo score of more than 1.2 were found. After a uniqueness check, 452 compounds remained which contained the five queries and database hits. The REINVENT model was pre-trained on these molecules for 35 epochs. Then the final production training on the docking score was run for 800 epochs, where the pre-trained model was used as prior and as agent.

The generated SMILES strings for all trainings were stored every 10th epoch. Those SMILES that could be converted to valid and unique chemical structures were used for further analysis that was performed analogously to the Lib-INVENT runs (see Section 2.4.2).

### 2.2 System for evaluation: Coagulation factor Xa

As example for the new design workflow we employed the serine protease factor Xa (fXa), as it is well-characterized in structural terms with many X-ray crystal structures in the PDB database and available structure-activity relationship (SAR) information (Bernstein et al., 1977; Burley et al., 2021). In particular, we focused on a representative X-ray structure for the indole-2-carboxamide series of fXa inhibitors (Figure 2). Here, the indole-2-carboxamide 1 (resolution 2.2 Å, PDB 2BOH, K_i_ 3 nM (Nazaré et al., 2005b)) was crystallized in complex with fXa and therefore allows identifying critical features for binding affinity (Nazaré et al., 2005b).

### 2.3 Reward functions to guide generative artificial intelligence-methods

#### 2.3.1 Ligand-based scoring functions

As a comparison of our design results to well-established methods, initial trainings were performed as a baseline where 3D similarity to the co-crystallized ligand was applied as scoring function (Grebner et al., 2020b). TanimotoCombo score as implemented in ROCS from OpenEye was employed as 3D similarity measure (Grant et al., 1996; Openeye Toolkits, 2019). Before using it in the scoring function for Lib-INVENT, it is scaled that values between 0.5 and 1.4 are projected linearly into the range between 0.01 and 1. Values below 0.5 are set to 0.01 and values above 1.4 are set to 1 (see Supplementary Figure S2). These thresholds were chosen to focus the learning on the most informative region of the score based on inhouse experience.

Furthermore, the previously reported (Grebner et al., 2020b) ligand-based QSAR model [based on Graph Convolutional networks implemented from the DeepChem library, version 2.2 (Github, 2019)] for predicting binding affinity (pK_i_) of fXa inhibitors was used as scoring function. This score is called fXa score. For more details, please refer to the original publication (Grebner et al., 2020b). Besides the model itself, which predicts the activity, this scoring function also considers an applicability domain estimate of the model, which is used as a binary score. We first calculate the similarity for the evaluated molecule with respect to the training data set of the model using a Morgan Fingerprint from RDKit (Rdkit, 2022). If the highest similarity is above 0.4, the molecule is considered to be similar enough to the training data sets and thus the model is assumed to be applicable. In contrast, if the highest similarity is below the threshold, the molecule is too dissimilar to the data and the score for these molecules is always set to zero. This approach is related to previous literature studies (Baringhaus et al., 2013), while the exact threshold value in combination with the chosen fingerprint descriptor was empirically derived. While this approach is a simplification of the complex topic of applicability of a model, it serves well in the current applications.

#### 2.3.2 Glide docking and scoring terms

For all scores using glide docking, we used a docking grid from the protein structure 2BOH from PDB which was prepared using the Schrödinger Protein Preparation Wizard with default parameters (Sastry et al., 2013). Compounds to be scored are first run through LigPrep, enumerating stereocenters, tautomers and protonation states (Schrödinger, 2020). They are then docked using Glide Extra Precision (XP) with flexible sampling (Halgren et al., 2004; Friesner et al., 2006). The crystallized ligand from PDB 2BOH serves as reference structure for MCS (maximum common substructure) core pattern comparison where the hydrogen bond including the backbone NH of Gly216 is constrained. The program Proplister from the Schrödinger Suite is used to obtain individual docking and scoring terms (Schrödinger Knowledge Base, 2018).

From the Glide-XP docking output, two different protein-ligand scoring terms were used: the Glide-gscore and the Glide-ligand_efficiency_sa score. The first term corresponds directly to the Glide-XP docking score without Epik state penalties (Friesner et al., 2006), while the latter term scales the original Glide-XP docking score by the number of heavy atoms to the power of 2/3 in order to approximate the effect of the molecular surface area (SA) (Friesner et al., 2006; Schrödinger Knowledge Base, 2021). The docking scores usually range from negative to positive values, however, positive docking scores do not have any physical meaning. Therefore, docking scores are transformed as follows before being applied in the Lib-INVENT workflow for maximizing the scores:

$$f\left(x\right)=\left\{ \begin{array}{cc} -x, & x<0\\ 0, & x\geq 0 \end{array}\right.$$ (1)

#### 2.3.3 New protein-ligand scoring function based on glide and RDKit terms

**FIGURE 3:**
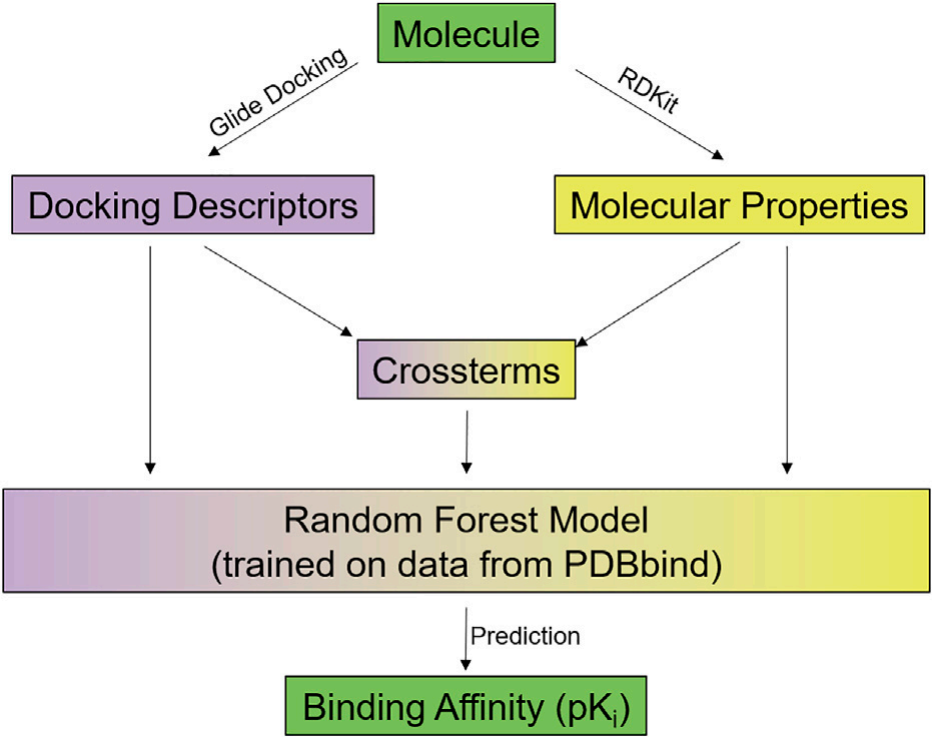
Overview of the RFXscore: Every molecule to be evaluated is docked using Glide and several docking descriptors are determined. Furthermore, a number of physicochemical properties is computed by RDKit. The docking descriptors, the RDKit properties, and some crossterms of both are put into a random forest model that predicts the binding affinity for the compound. Further details see text and Supporting Information.

In addition, a newly derived protein-ligand scoring function was used, which integrates individual Glide XP scoring terms as structure-based information with ligand-based 2D descriptors computed using RDKit into a predictive statistical model (see Figure 3).

For each protein-ligand complex, a total of 26 descriptors are extracted from Glide-XP (see Supplementary Table S1). These include individual energetic terms (e.g., total energy, Van der Waals, Coulomb), hydrogen-bond terms, different description of hydrophobic enclosures (Friesner et al., 2006), penalties for atoms located in unfavorable environments, and terms for less common protein-ligand interactions like π-cation interactions.

In addition to these structure-based terms, the ligands are characterized by a total of 40 2D descriptors taken from RDKit (see Supplementary Table S2) (Rdkit, 2022). This includes properties like number of heavy or hetero atoms, number of hydrogen bond donors/acceptors, logP, TPSA, number of rings, and MOE-type subdivided surface area descriptors using partial charges or logP contributions (Labute, 2000).

Furthermore, 42 cross-terms are computed as quotients of Glide descriptors and RDKit properties (see Supplementary Table S3). The goal here is to individually scale the Glide energy term contributions by specific features of the ligand in the binding site. From Glide, the following terms were used to compute these cross-terms: GlideScore, van der Waals energy, Coulomb energy, modified Coulomb - van der Waals interaction energy, H-bond term, and lipophilicity term. Each of these individual terms is divided by the following RDKit terms, namely number of heavy atoms, number of hetero atoms, logP, TPSA, fraction of C.sp3, N and O count, NH and OH count, which finally results in the 42 cross-terms (6 glide-terms * 7 RDKit-terms).

The final protein-ligand scoring model was then trained from the “refined set” of protein-ligand complexes from the PDBbind 2019 database (Wang et al., 2004; Liu et al., 2017; Wang, 2020). This set is compiled from the general PDBbind set and contains complexes with better experimental quality along with experimental binding affinity and a converted ligand file with validated atom typing. Furthermore, a number of filters regarding binding data, crystal structures, as well as the nature of the complexes were applied (Liu et al., 2017). The refined set in the 2019 release contained 4,852 protein-ligand complexes. Each input PDB file was processed using the Protein Preparation Wizard using default settings in an automated workflow. It was assumed that protonation state and geometry is acceptable in the refined set, which we confirmed by visual inspection in several complexes. In addition, crystallographic water molecules were deleted, and disulfide bridges were formed.

As next step, a Glide grid file for each complex was automatically generated using standard settings without constraints. For each successfully converted complex, a Glide XP scoring step without altering the ligand geometry was performed in order to obtain the Glide and XP terms as input. Some complexes could not be successfully processed, or not all descriptors could be computed. Those were rejected, which resulted in a final training set of 4,231 complexes from the PDBbind set for further scoring function development.

This set then was split into a training set of 3,591 compounds and a test set of 640 compounds. To assure a balanced activity distribution between both sets, the compounds were first partitioned into 10 evenly distributed pK_i_ activity bins and 15% were randomly selected from each bin as test set. A random forest (RF) model (Breiman, 2001) was developed to correlate descriptors with the experimental affinity, expressed as pK_i_ or pIC_50_ values. The model was generated with scikit-learn (Pedregosa et al., 2011) using 500 individual trees and the mean absolute error (MAE) as criterion for optimization. A 10-fold cross-validation strategy in 3 repeats each served as validation approach for the training set. A significant model with a cross-validated *r* ^2^ value of 0.459 (maximum *r* ^2^ 0.545, StdDev: 0.047) and an *r* ^2^ value of 0.855 resulted with a MAE of 0.578. For the independent test set, a predictive *r* ^2^ of 0.496 was obtained (MAE: 1.121).

In addition to this PDBbind training and test set, we added numerous internal X-ray structures and binding affinity data for two internal projects, namely factor Xa (see above) and Renin (Scheiper et al., 2010; Matter et al., 2011). In all cases, resolutions for added structures are <3.0 Å, while structures with a resolution <2.5 Å are preferrable. For factor Xa, 10 X-ray structures representing two main series, namely indole-2-carboxamides and oxybenzamides were added. A representative structure for the indole-2-carboxamide series is found in the PDB file 2BOH (resolution 2.20 Å) (Nazaré et al., 2005b). For the oxybenzamide series, the PDB file 2BMG provides a typical example (resolution 2.70 Å) (Matter et al., 2005). For Renin, 8 X-ray structures representing two series of potent analogs are added to the dataset. Here, the PDB file 3OOT (resolution 2.55 Å) provides a typical example for the indole-3-carboxamide series (Scheiper et al., 2010). In addition, X-ray structures for analogs of the inhibitor Aliskiren were added, as exemplified by the PDB file 2V0Z (resolution 2.20 Å) for this prototypical Renin inhibitor reported by Rahuel et al. (2000)

**FIGURE 4:**
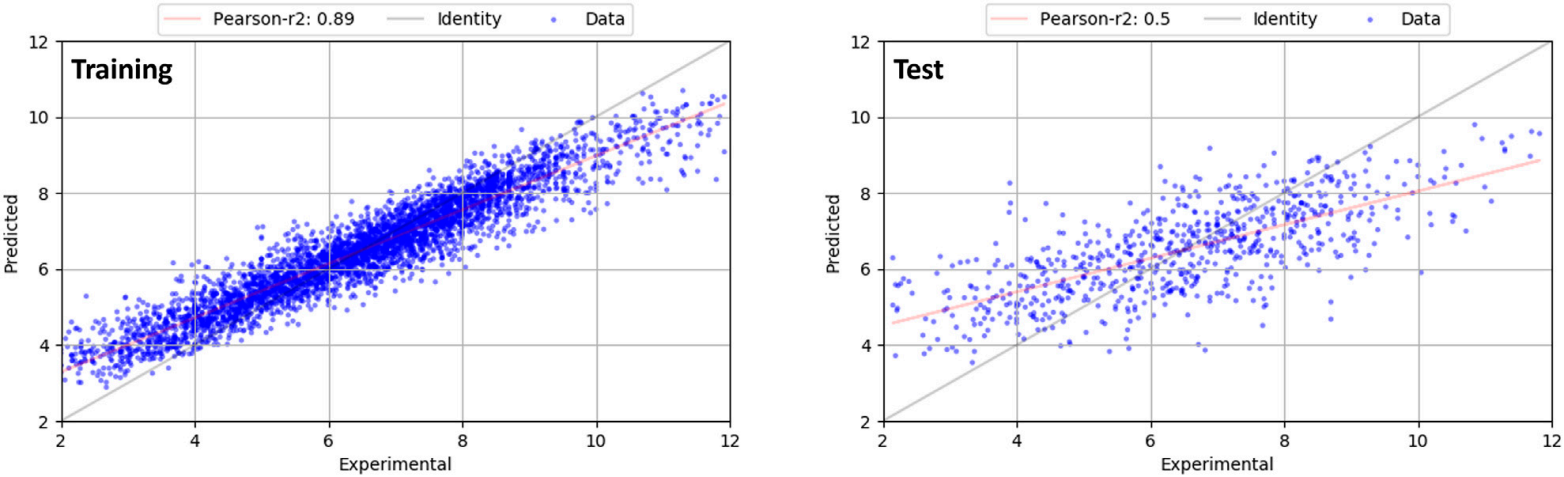
Graph of experimental pK_i_ values on the *x*-axis *versus* predictions from the final random forest model (*y*-axis) for the training (left) and test dataset (right). Line of identity is shown in grey.

We further augmented this dataset by reliable binding poses from carefully docked and inspected factor Xa and Renin inhibitors as close analogs to the internal X-ray structures. All the additional data was added to the training set, so this resulted in an updated training set of 3,995 compounds and 719 compounds in the test set. With this updated dataset, a final model with a cross-validated *r* ^2^ value of 0.460 (maximum *r* ^2^ 0.533, StdDev: 0.037) and an *r* ^2^ value of 0.856 resulted with a MAE of 0.556. For the test set, a predictive *r* ^2^ of 0.494 was obtained (MAE: 1.086). The graph of experimental pK_i_ values on the *x*-axis *versus* predictions from this model (*y*-axis) is shown in Figure 4 for the training (left) and test dataset (right). While the statistics of this model remains similar to the general new protein-ligand scoring function, it focuses the chemical space for the target proteins used in this *de novo* design study. Therefore, this model was used throughout the entire study in this manuscript. pK_i_ predictions using both models are scaled in a similar manner as those from the QSAR model.

Random forest models are often used as robust approach for high-dimensional regression. They require little hyperparameter tuning and have only a low probability for overfitting. In this method, predictions from an ensemble of decision trees are finally averaged for an overall predicted value. This averaging could introduce a systematic bias of the resulting models. In fact, it is reported that models can be sometimes too conservative, i.e., predictions of extreme values are shifted towards the mean value of the dataset (Zhang and Lu, 2012; Hooker and Mentch, 2018). We also observe this in our models, which is exemplified by the difference of the slope of the regression line (red) compared to the line of identity (grey) in Figure 4, in particular for the test set. Hence, this model systematically over- or under-predicts at either end of the plot for the test set, while the relative ranking is preserved. However, as we did not obtain significantly better models using other methods (PLS, regression trees), we maintained the final random forest model.

While no significant outliers were observed for the training set, larger deviations were found for the test set, as seen in Figure 4 (right panel). For the factor Xa or Renin series, no significant outlier predictions are observed. Furthermore, a list of main outliers for the test set and a further discussion is given in the Supporting Information.

#### 2.3.4 Estimation of druglikeness

In additional runs, the RFXscore was combined with a score for the “Quantitative Estimate of Druglikeness” (QED) and with a QSAR model predicting pK_i_ values against fXa (fXa model) (Bickerton et al., 2012; Grebner et al., 2020b).

The QED is a number between 0 and 1 which describes the “druglikeness” of a molecule by comparing its physicochemical properties to their distribution in a set of approved drugs (Bickerton et al., 2012). It is computed as the weighted geometric mean of several so-called desirability functions d_i_:

$$QED=\exp\left(\frac{\sum_{i=1}^{n}w_{i}\;d_{i}}{\sum_{i=1}^{n}d_{i}}\right)$$ (2)

Each desirability function d_i_ corresponds to one of the following molecular properties: Molecular weight, octanol-water partition coefficient, number of hydrogen bond donors, number of hydrogen bond acceptors, molecular polar surface area, number of rotatable bonds, number of aromatic rings, and number of structural alerts (Bickerton et al., 2012).

In our scoring functions, QED was computed *via* RDKit and taken “as is”. The fXa score was obtained and scaled like described in Section 2.3.1. The total score was calculated as the arithmetic mean of the two single scores, i.e., RFXscore + QED or RFXscore + fXa.

#### 2.3.5 Glide-ROCS

In order to speed up the computation for individual design runs, we implemented an option to initially perform a 3D shape overlay with the reference ligand and use the resulting conformer as a starting point for refinement docking, in analogy to Kelley et al. (2015). The 3D shape overlay is performed using ROCS from OpenEye by maximizing the Tanimoto Combo similarity (Grant et al., 1996; Openeye Toolkits, 2019). In order to prepare it for docking, hydrogens are added to the “best” conformer *via* the OpenEye function OEAddExplicitHydrogens() before writing the structure into an sd-file (Openeye Toolkits, 2019). This file is now directly put into the docking process with docking method *mininplace*, which means that no ligand sampling is performed but only a local optimization of the ligand (Chaput and Mouawad, 2017). Unlike before, no reference ligand is used and no constraints are set. The docking output needed is again obtained using Proplister and postprocessing is performed like described in the previous section.

### 2.4 Model evaluation

For evaluating the different generative design runs, two important aspects were considered: The first is the training performance, i.e., how successful the model learns to generate suitable compounds based on the applied scoring function. The second aspect is to analyze the diversity and usefulness of the generated molecules themselves. As the scoring function is different for every run, other metrics to judge the quality of the compounds should also be considered.

**FIGURE 5:**
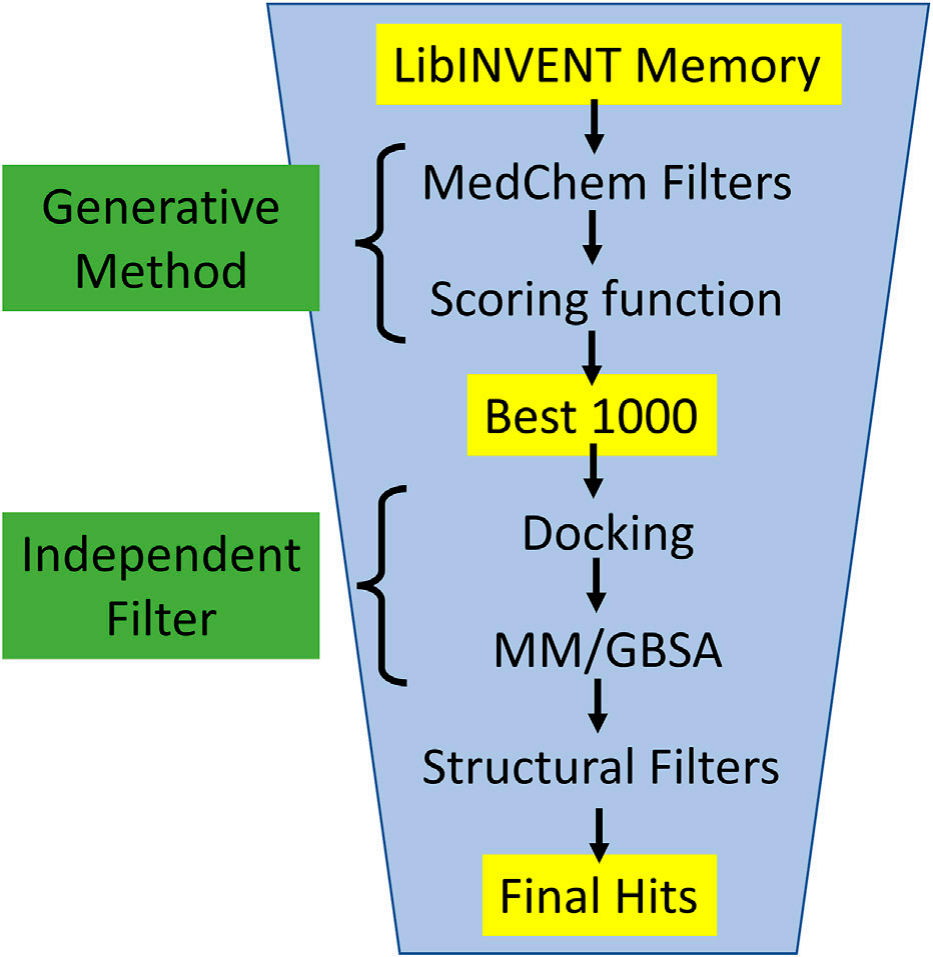
Compound evaluation workflow: All generated molecules from memory are filtered based on physicochemical properties to enforce the generated compounds to be inside a scope that is generally considered as drug-like (MedChem). Then the 1,000 remaining compounds with the highest scores are re-docked and undergo an MM/GBSA analysis. Only compounds with a MM/GBSA energy of less than −70 kcal/mol are considered further. In the last step, several structural filters are applied to remove compounds with undesired substructures from the final hits.

In this work, we applied several filtering steps to evaluate the generated molecules (see Figure 5): Starting from the entire Lib-INVENT memory (i.e., all compounds with a score >0.4), or all sampled molecules from REINVENT, we first apply filters on physicochemical properties to remove compounds that are not considered “drug-like” (MedChem filters). The 1,000 highest-scoring molecules are then selected for the next step which serves as an evaluation procedure independent of the generation method. The compounds are redocked and Molecular Mechanical/Generalized Born Surface Area (MM/GBSA) (Greenidge et al., 2013) is applied. The free energy computed by MM/GBSA can be seen as a selection property that is not biased, neither by the scoring function of the generator nor by human prejudice. In the last step, the compounds with a low MM/GBSA energy are run through several structural filters that remove substructures that are generally not desired for drug-like compounds.

#### 2.4.1 Evaluation of training performance

In order to evaluate the training progression, the average score, resulting from the employed scoring function, for all generated molecules was plotted for each epoch. For Lib-INVENT, plotting is performed for scores with and without application of the diversity filter. As the diversity filter is applied before reinforcement learning, the score including them shows the success of the training itself, while the “pure score” without diversity filter (explanation see above) indicates the quality of the generated molecules, where quality is measured by the current score. Furthermore, by comparing the two curves it is possible to estimate the diversity of the compounds generated during training.

As another metric, we computed the percentage of valid, unique, and novel molecules during each training epoch (Brown et al., 2019). A SMILES string is defined as valid if it can be converted to a molecule by the RDKit suite. A molecule is counted as unique if it has not appeared in any of the previous training epochs, whereas it is novel if it was not present in the training set for the prior (Brown et al., 2019). Identical molecules are detected by comparing the canonical SMILES strings using RDKit (Rdkit, 2022).

#### 2.4.2 Evaluation of generated molecules

For the evaluation of the generated molecules in Lib-INVENT, the yield is suggested as a metric to evaluate the degree of success of the runs (Fialkova et al., 2021). It is defined as the number of molecules in memory divided by the number of all generated molecules (Fialkova et al., 2021):

$$yield=\frac{\left|Scaffold\;memory\right|}{Batch\;size\times Number\;of\;steps}$$ (3)

As the yield corresponds to the fraction of unique molecules with a score above the cutoff of 0.4, it combines information about how many different compounds are generated and how good they are in the scope of the scoring function. Additionally, we defined some filters based on physicochemical properties to enforce the generated compounds to be inside a scope that is generally considered as drug-like. Specifically, only molecules were kept where the following conditions are fulfilled:

• Molecular weight between 250 and 550 Da.
• Polar Surface Area (PSA) between 50 and 150.
• Number of heavy atoms between 20 and 50.
• Number of rotatable bonds not bigger than 10.
• Number of hydrogen bond acceptors between 1 and 10.
• Number of hydrogen bond donors between 1 and 5.

Out of the filtered molecules, the 1,000 highest scoring compounds have been extracted for each Lib-INVENT run.

For all 13,000 compounds combining all runs, the t-distributed stochastic neighbor embedding (tSNE) was calculated to evaluate the chemical distribution and diversity of the generated molecules (Maaten and Hinton, 2008). The tSNE was computed as follows: For all molecules, Morgan fingerprints with radius 2 were created by RDKit (Rdkit, 2022). Then, a Principal Component Analysis (PCA) was performed on them (Wold et al., 1987). The 50 most important components from PCA were used in the tSNE algorithm to project compounds into two dimensions where they can easily be plotted. PCA and tSNE were both performed using scikit-learn (Pedregosa et al., 2011). The same tSNE coordinates were used for all plots to ensure that the position of a particular molecule in different plots is preserved.

For the next step, the molecules were docked into the grid created from 2BOH with the same parameters as in the scoring function Glide-gscore. The MM/GBSA approach, as implemented in Schrödinger/Prime was applied on the 250 structures with the lowest docking score for each Lib-INVENT run (Greenidge et al., 2013). The OPLS3e force field was employed and protein residues within 3 Å around the ligand were treated as flexible in Prime. All ligands with a computed free energy below the threshold of −70 kcal/mol (10% above the energy of the reference ligand) were then selected for further analyses.

In the last step, compounds with undesired substructures as described above were removed (Baell and Holloway, 2010). Specifically, the molecules should contain no more than one hydroxy or primary amine group, no anilines or phenols, and no aliphatic bonds between the heteroatoms N, S, and O. An internal list of substructure filters was applied to eliminate molecules with presumably non-druglike, reactive and toxic fragments. This list is based on an internally reviewed collection of public domain substructure filters, as exemplified in the following references (Rishton, 1997; Baell and Holloway, 2010). The remaining molecules passing the filters were projected into the former tSNE embedding.

For molecules from REINVENT runs, the entire filtering workflow remains identical.

## 3 Results and discussion

Two methods for generating molecules have been analyzed: REINVENT and Lib-INVENT. Results are first described for Lib-INVENT, where all the different scoring schemes have been tested. Then, the two most promising scoring functions have been applied to REINVENT with and without transfer learning. In general, results about the performance of scoring functions should be transferable between REINVENT and Lib-INVENT, as the general reinforcement strategy for generative design remains similar (Fialkova et al., 2021).

### 3.1 Lib-INVENT computations

#### 3.1.1 Model performance

**FIGURE 6:**
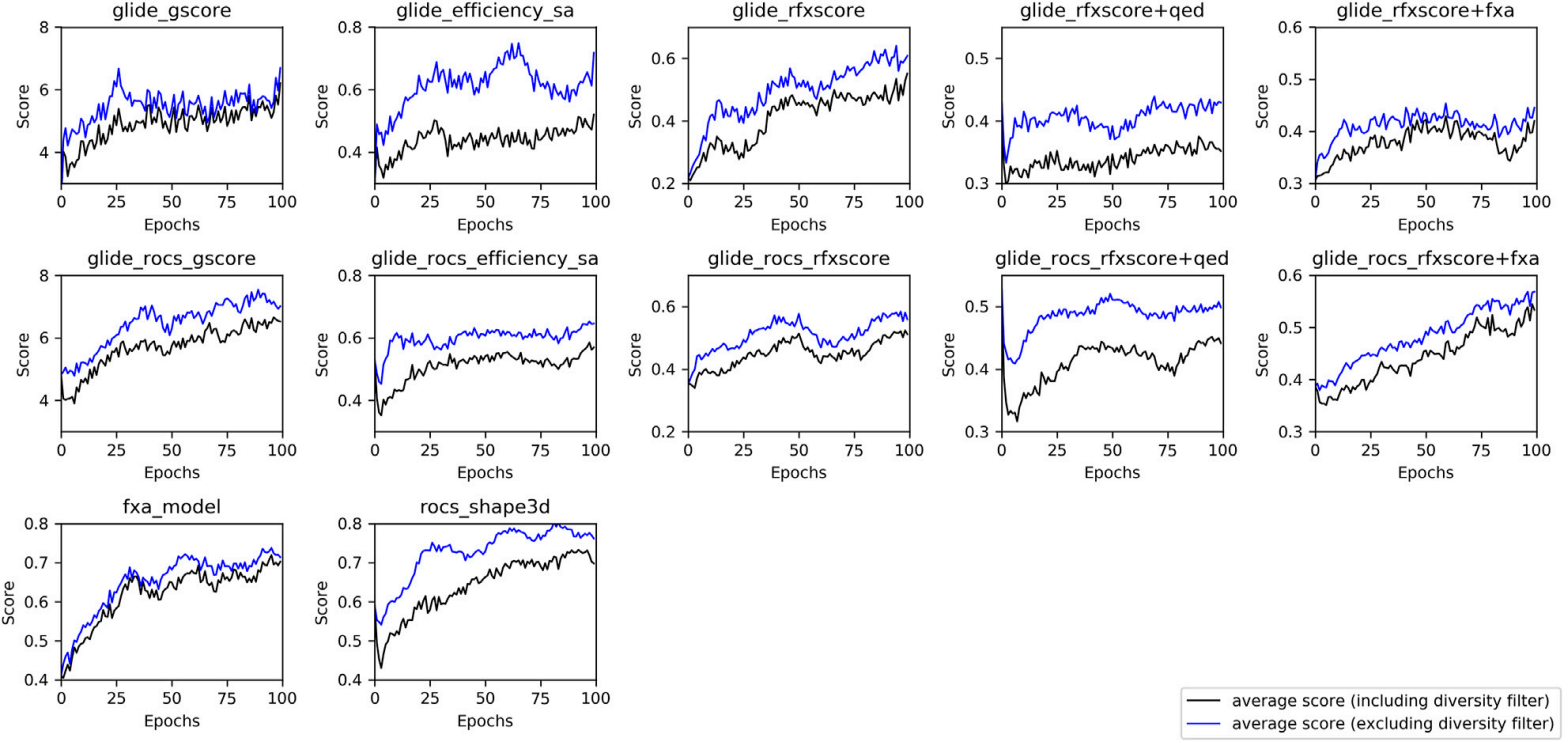
Average score from one run for each method with a batch-size of 256 molecules per epoch during Lib-INVENT training (black: including diversity filter, blue: score without diversity filter).

In order to investigate whether the optimization procedure was successful, the average score during training was plotted for each run (see Figure 6). In this context, the average score corresponds to the score resulting from the respective scoring function, i.e., the underlying methods are different for each run and thus cannot be compared directly. In addition, gscore and ligand_efficiency_sa describe absolute values, the other scores are projected into a range between 0 and 1 (see Section 2.3.2).

Most of the training runs reached a plateau of the score within 100 epochs. The learning success for the combined scores Glide-RFXscore + QED and Glide-RFXscore + fXa is rather poor, although both RFXscore and the fXa model show a good training performance when used alone. This happens when the two scores trained simultaneously are negatively correlated to each other, as it is the case for RFXscore and QED (see Supplementary Figure S3). This negative correlation is mediated by the polarity of the molecules which is shown as the number of possible hydrogen bonds (sum of H-bond-donors and H-bond-acceptors). Since the protein pocket is quite polar, the RFXscore favors compounds with a lot of these groups (around 8–11), whereas the definition of the QED prefers lower numbers (around 4–7). The upper right corner, where molecules are found for which both of the scores are in the desired range, is not populated, so the model will not be able to move here.

As can be seen in Supplementary Figure S4, all methods maintain high ratios of valid and novel compounds (>90%) and a sufficiently high ratio of unique compounds that ranges from around 90% for the fXa model to around 50% for Glide-ligand_efficiency_sa.

As the average score for the ensemble of generated molecules does not necessarily correlate to the final quality of generated molecules, we now took the ensembles and performed further evaluation.

#### 3.1.2 Generated molecules

As described in Section 2.4, the generated molecules were filtered in a three-step process (see Figure 5). In the first step, the scores from the generative runs were applied to select 1,000 compounds. Then, the MM/GBSA energy served as an independent judge to compare the different engines. At last, those molecules passing the MM/GBSA threshold were subjected to a structural filter to remove undesired molecules. This workflow represents a typical flow applied in a structure-based drug design project. Therefore, this gives a reasonable judgement about the quality and usefulness of molecules generated by the different approaches.

**FIGURE 7:**
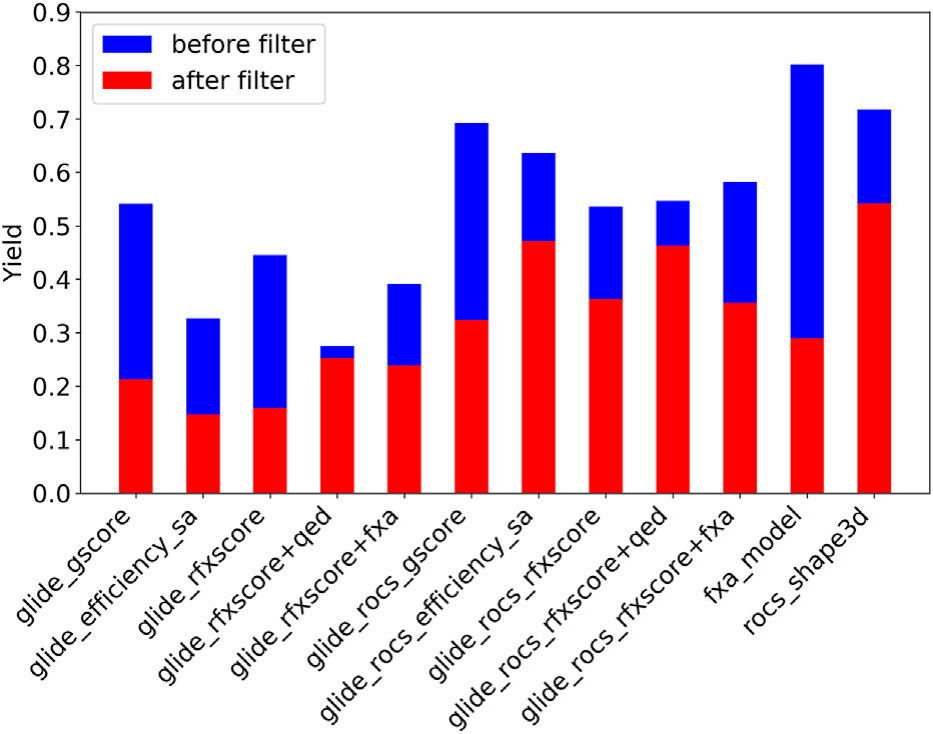
Yield for Lib-INVENT runs with different scoring functions. MedChem filters were applied on scaffold memory.

Figure 7 shows the Lib-INVENT yield in blue, i.e., the ratio of unique compounds with a score >0.4 that are generated during the training. It is highest for the fXa QSAR model and the 3D similarity, and lowest for Glide-RFXscore + QED. Glide-ROCS results in higher yields than pure Glide, leading to the conclusion that it might be easier for the engine to fill the pocket if the orientation of the compound is already pre-determined by the 3D overlay. After applying the physicochemical property filters (see Figure 7, red bars), most molecules remain for the 3D similarity, Glide-ROCS-ligand_efficiency_sa, and Glide-ROCS-RFXscore + QED, while more than half of the molecules in scaffold memory are filtered out for the fXa model, Glide(-ROCS)-gscore and Glide-RFXscore. When the QED score is included into the scoring function (Glide-RFXscore + QED, Glide-ROCS-RFXscore + QED), only very few compounds are removed, which shows that the properties chosen as druglikeness conditions are in good agreement with this estimate of druglikeness.

To compare the quality of the compounds generated by the different methods, the 1,000 highest scoring molecules after filtering were extracted for each method as described above.

**FIGURE 8:**
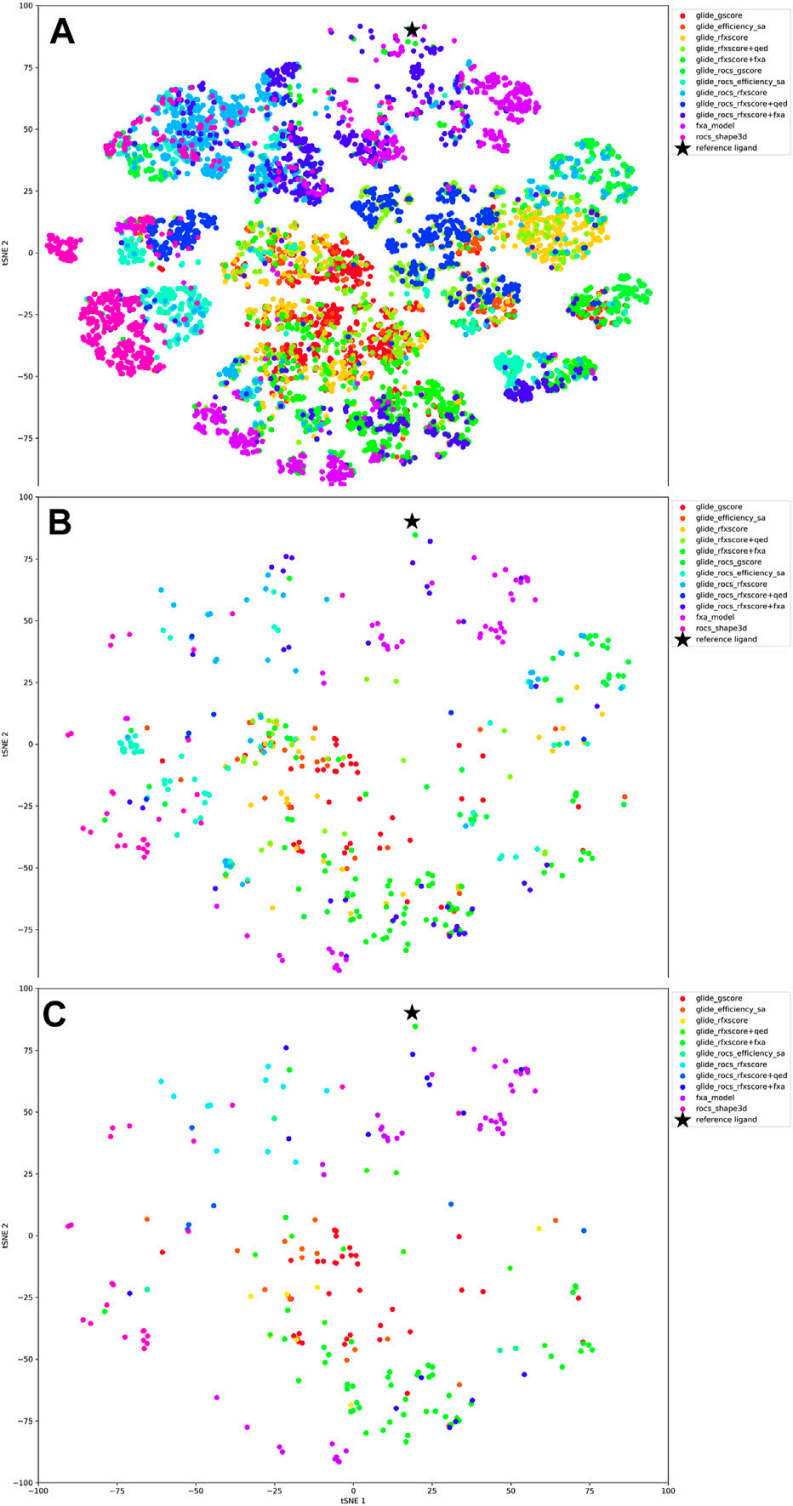
tSNE plots, color-coded according to the scoring function. **(A)** all 1000 extracted molecules for each run, **(B)** molecules after MM/GBSA, **(C)** finally selected molecules.

The tSNE plot of all 13,000 compounds (see Figure 8A) shows that different scoring methods explore different regions of chemical space, although there is substantial overlap. The application of a free energy cutoff after MM/GBSA analysis clearly reduces the number of molecules but does not considerably reduce the compound diversity as obvious from their broad distribution in the tSNE plot (Figure 8B).

**FIGURE 9:**
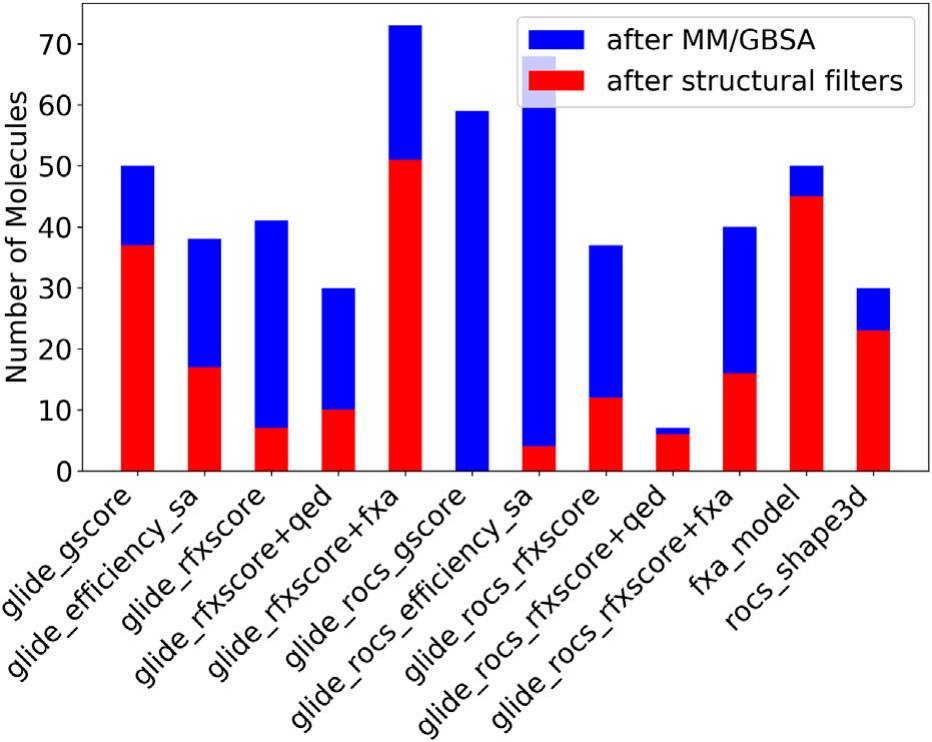
Number of molecules remaining after filtering by MM/GBSA (blue + red) and unwanted fragments (red).

After this step, most compounds remained for Glide-RFXscore + fXa which combines the structure-based scoring function with a pure ligand-based QSAR model (see Figure 9, blue bars). The Glide-ROCS scores (gscore, ligand_efficiency_sa, and RFXscore) produce a higher number of molecules with good MM/GBSA scores than the corresponding Glide-only scores. The pre-alignment facilitates the generation of molecules fitting the binding site resulting in a better binding free energy. Combining the RFXscore with QED results in a smaller number of remaining compounds (independently of the application of ROCS pre-alignment), while adding the fXa QSAR model increases the number of compounds with good MM/GBSA energy.

After filtering undesirable structural motifs, the biggest number of molecules remains for the combined score of RFXscore and QSAR model (see Figure 9, red bars). A large number of compounds also remains for the pure fXa model and for the “normal” Glide-gscore. RFXscore alone, however, produces only very few compounds that pass all the filters. Most unwanted fragments are found in the molecules generated with the ROCS pre-alignment, which is mainly due to multiple polar groups like OH or NH_2_. However, the ratio of accepted molecules increases when applying RFXscore or even RFXscore + fXa instead of Glide-gscore.

Looking at the tSNE plot in Figure 8C, it can be seen that the compounds that were created by the QSAR model are quite close to the reference molecule. This is not a surprise as the scoring function as well as the tSNE plot are based on 2D representations of molecules. Compounds generated with the docking scores Glide-gscore and RFXscore + fXa are much more diverse and less similar to the original inhibitor. Interestingly, the results from the Glide-ROCS runs, spread over nearly the whole chemical space, although their number is comparably low.

In summary, the results show that the tailored structure-based scoring function RFXscore together with a QSAR-model results in the most promising structures which are also structurally different to the reference compounds. Some examples for docking poses of molecules generated with this scoring function are shown in Supplementary Figure S5. A pure 2D-based QSAR model scoring also results in a large number of acceptable molecules, however, those molecules are very close analogs to the reference (as expected). This also highlights that the average score discussed in the beginning of this section does not necessarily correlate to the final quality of molecules. Most importantly, the average score as a metric should only indicate if there is a learning progress in the method.

As already stated in the introduction and goal setting, the chosen approach of combining structure-based-scoring with Lib-INVENT is mainly intended for lead optimization with focus on R-group replacement, thus a rather focused search for new molecules. Extension of these scoring schemes to other generative engines such as REINVENT can offer ways to vary molecules more broadly towards a lead finding scenario, which will be evaluated in the next section.

### 3.2 REINVENT computations

To evaluate REINVENT as an example for lead generation, we used the most promising scoring functions from the Lib-INVENT runs to start REINVENT computations for 1,600 epochs. Based on the results of the last section, we chose RFXscore and RFXscore + fXa, as the RFXscore + fXa score gave the best results and RFXscore gives a direct comparison about the effect of including the fXa model. To access a potential acceleration of the generation runs, we also explored a transfer-learning pre-training using a model focused on 452 molecules with a high 3D similarity to known fXa drugs. The generated SMILES strings were stored every 10th epoch.

**FIGURE 10:**
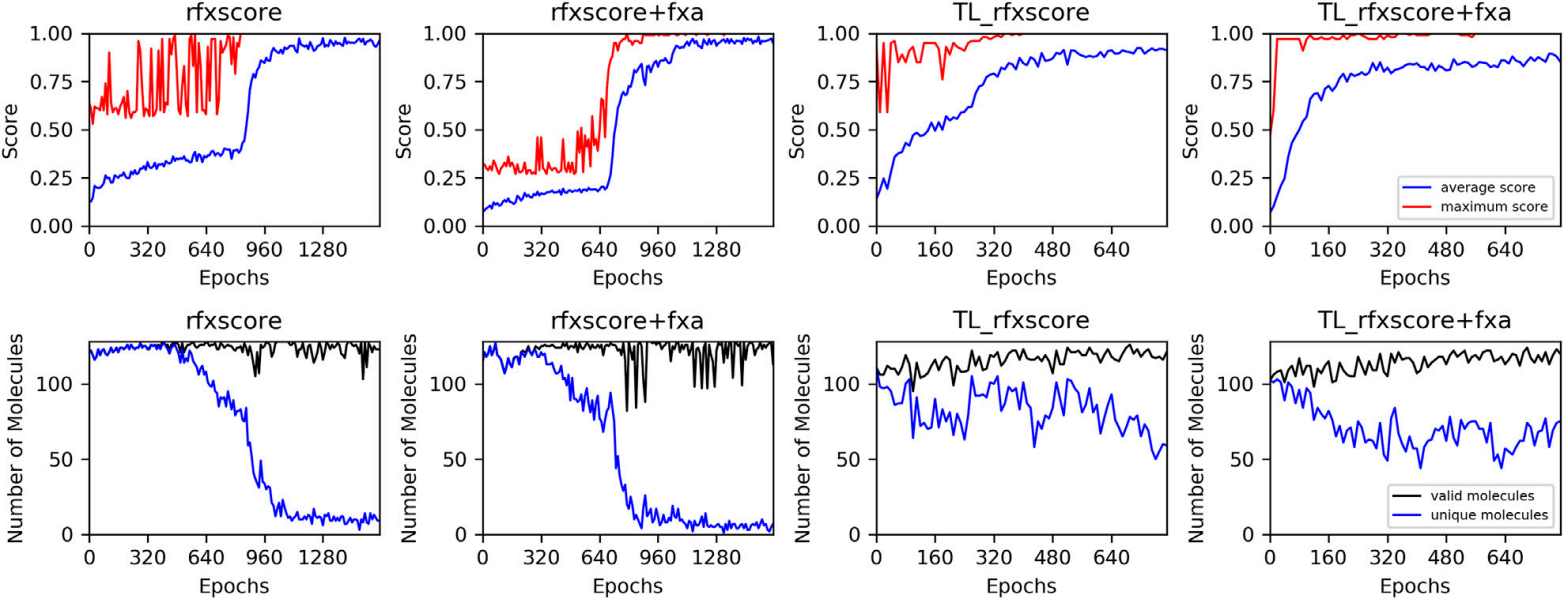
Training performance of REINVENT runs. Upper row: average (blue) and maximum (red) score, lower row: number of valid (black) and unique (blue) generated molecules.

The plots of the average score during these runs (Figure 10, upper row) show that convergence is reached after around 800 epochs without and 300 epochs with transfer learning at an average score of over 0.8. This clearly demonstrates that the generation runs can be accelerated very efficiently with transfer learning pre-training. In the second row, the number of valid and unique molecules is shown. A molecule is counted as unique if it was not present in any former sample. From 128 generated SMILES per epoch, the number of valid molecules never drops below 100 for any of the runs. The number of unique molecules, however, suddenly drops close to zero for the runs without transfer learning when the score reaches its plateau. This indicates that the optimization in these runs gets stuck in a local minimum and generates the same high-scoring molecules repeatedly. For the runs with transfer learning, the number of unique molecules also decreases but much slower, so there are still new compounds created in the final epochs. This might be explained by the fact that the model is already focused by transfer learning towards regions of chemical space where an exploitation by docking scores finds different acceptable molecules, whereas a model trained on a huge database consisting of very diverse molecules only very rarely finds compounds with a good score, which makes it easier to get stuck at one of them.

**FIGURE 11:**
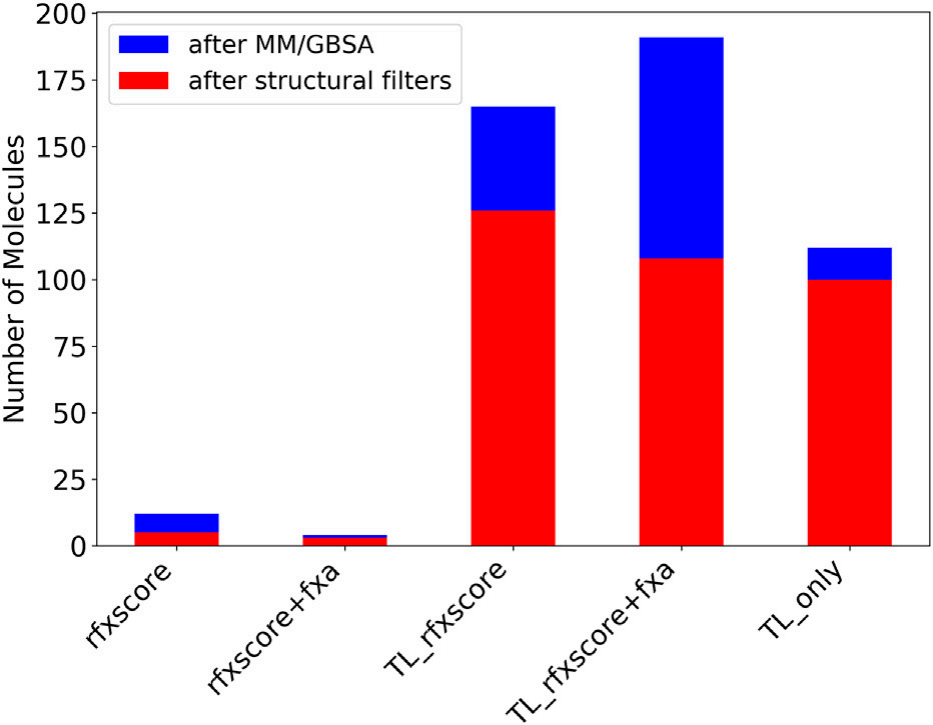
Number of molecules after MM/GBSA (blue + red) and structural filters (red).

After applying MedChem filters, the 1,000 best-scoring virtual hits were docked into the fXa binding pocket and submitted to MM/GBSA like already described for Lib-INVENT runs. Molecules with a score <−70 kcal/mol (plus 10% tolerance compared to the reference 2BOH with −77.42 kcal/mol in this run) were kept for further analysis. The blue bars in Figure 11 show the number of compounds remaining after this step. The last bar (TL_only) was created by just sampling 1,000 SMILES strings from the pre-trained model and running them through the same filtering workflow. The RL runs with pre-training from transfer learning produce significantly more compounds with a good MM/GBSA score than those without transfer learning. The number of accepted compounds is even higher than that obtained only by transfer learning. This illustrates that the RL-optimization with the two scoring functions is able to further optimize the focused transfer learning prior.

After that, the same structural filters as for the Lib-INVENT runs were applied. The number of remaining molecules is depicted in red in Figure 11. It is biggest for the TL_RFXscore run with around 125 compounds. More molecules are removed here for the run with the combined score of RFXscore and QSAR model, but still more than 100 compounds remain. This is slightly more than for the computation where SMILES were just sampled from the pre-trained model. Here, only very few compounds are removed in this step, probably because the model was trained on a compound set that didn’t contain any unwanted structural motifs.

**FIGURE 12:**
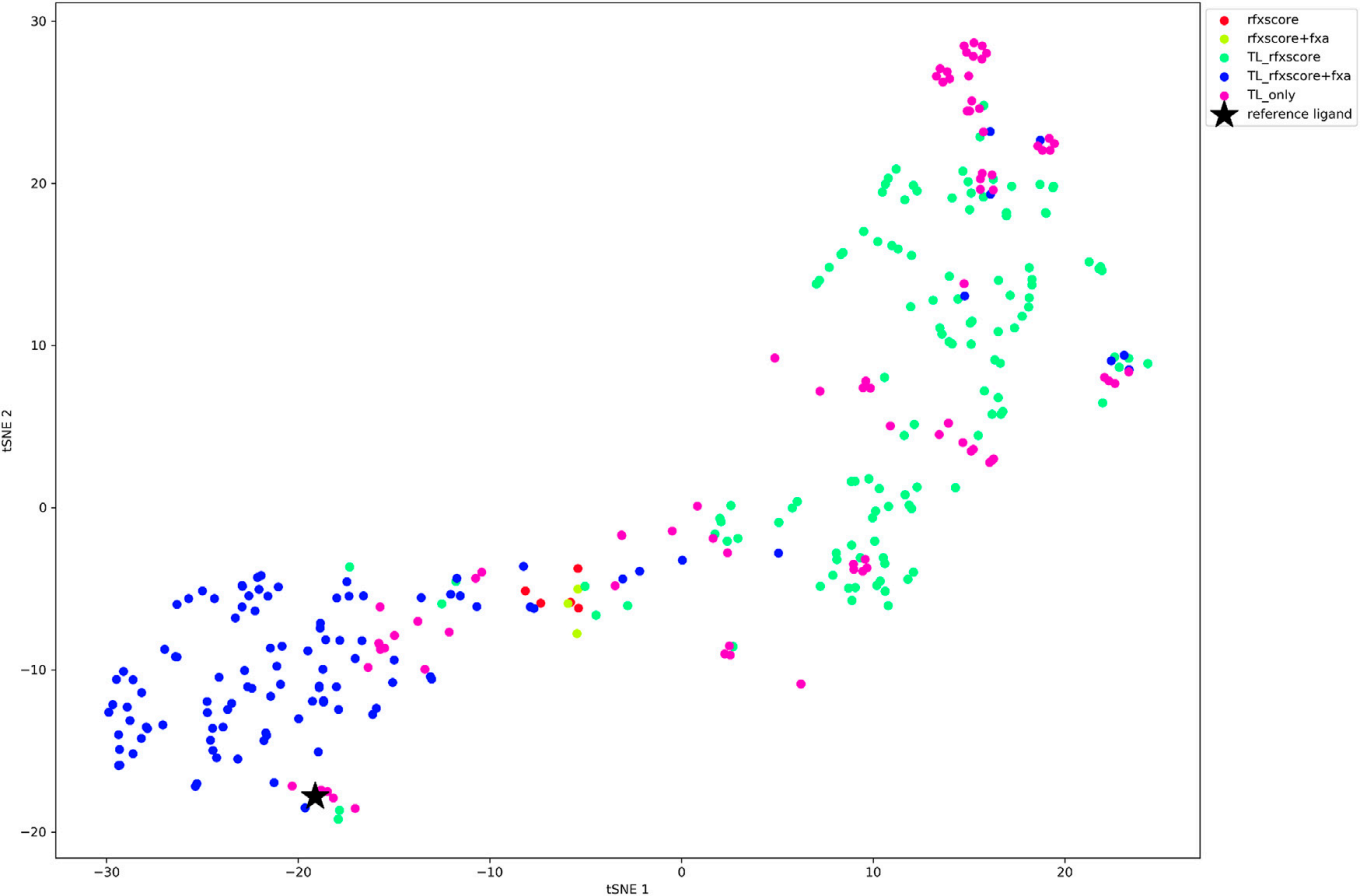
tSNE plot of the final molecules for each REINVENT run, color-coded according to the scoring function.

When looking at the tSNE plot of the finally accepted molecules (Figure 12), it can be stated that RFXscore and the combined score RFXscore + fXa cover different regions of chemical space, where the compounds generated including the QSAR model are closer to the reference structure. The structures from pure transfer learning populate small islands around the reference inhibitor as well as at other spots due to the presence of novel chemical series used for transfer learning from ChEMBL.

The finding that different scoring functions cover different regions of chemical space is supported by directly comparing the generated molecules. The Venn diagram of the three transfer learning runs shows only very small overlap between the compound sets (see Supplementary Figure S6). The sets from the two runs without transfer learning do not overlap at all, neither to each other nor to one of the transfer learning runs.

These results show that the RFXscore scoring function is able not only to generate promising new compounds in a lead optimization scenario (Lib-INVENT), but it can also find interesting molecules in a lead finding scenario without structural restrictions (REINVENT). The convergence of the engine as well as the number of acceptable output molecules can be improved by pre-training and consecutive transfer learning.

## 4 Conclusion

In the present study, we explored different 3D-structure based scoring functions for generative *de novo* design methods and compared the results to the baseline of 3D-similarity and QSAR-model based scoring. We evaluated two different methods, the REINVENT framework based on RNN-SMILES to sample the entire chemical space and the Lib-INVENT framework for a more focused exploration. As convergence was found to be slower with REINVENT combined with significantly higher computational demands, we focused the general comparison of scoring schemes on Lib-INVENT first, where we envision the results to be transferable to other generative AI-methods as well. This transferability is illustrated by the REINVENT test runs with the most promising scoring functions found in the Lib-INVENT study. Thus, the present study can be of potential value for structure-based AI-*de novo* design.

In summary, a large number of molecules accepted in the last step originates from the ligand-based scoring function (i.e., QSAR-model). However, this result appears to be quite obvious as the ligand-based scoring delivers more similar molecules and thus stays close to the reference structure. This approach is therefore very valuable in a focused lead optimization scenario where only close analogs are desired.

If the main goal is lead optimization with more diverse molecules, i.e., varying R-groups, or replacement of parts of the molecule, the structure-based scoring schemes together with Lib-INVENT deliver very promising results. The most compounds passing all filters are obtained when docking poses are scored using the RFXscore method in combination with the QSAR-activity model for the target. As this combination has shown to be quite effective, this might suggest an avenue for combining 3D with 2D scoring schemes for generation.

In a lead finding scenario, which we conducted using REINVENT, RFXscore alone resulted in more acceptable compounds than the combination of RFXscore and QSAR, but the number of molecules was in general very low and the computations very slow. In order to improve the results with respect to quality and computational performance, we recommend using transfer-learning with a model pre-trained from a diverse set of starting structures with high 3D similarity to known inhibitors. This transfer learning step not only sped up the calculation significantly (less than half epochs needed until convergence), but also increased the number of acceptable compounds at the end of the filtering process. As can be seen in Figure 12, RFXscore alone results in more diverse and potentially novel compounds while the combined score performs a more local exploration, which allows to tune the generation into the desired direction.

In summary, structure-based scoring indeed delivers novel and high-scoring molecules. Those are close analogs to known inhibitors as well as more diverse compounds. The combination of docking scores with other metrics is quite effective to bias the generation of novel and relevant structures into desired chemical space. Furthermore, the combination with a transfer learning step allows to accelerate and improve the results significantly.

## Data Availability

The full datasets include corporate and confidential molecules and cannot be published as is. Data from PDBbind is available publicly. Requests should be directed to Christoph Grebner, christoph.grebner@sanofi.com.
