# Supplementary material for: Optimizing interactions to protein binding sites by integrating docking-scoring strategies into generative AI methods
Source: Front Chem. 2022 Oct 19;10:1012507. doi: 10.3389/fchem.2022.1012507 (PMC9629386; doi:10.3389/fchem.2022.1012507)
Supplement: Supplementary file 1 [file DataSheet1.PDF]

Supplementary Material

1 Supplementary Figures

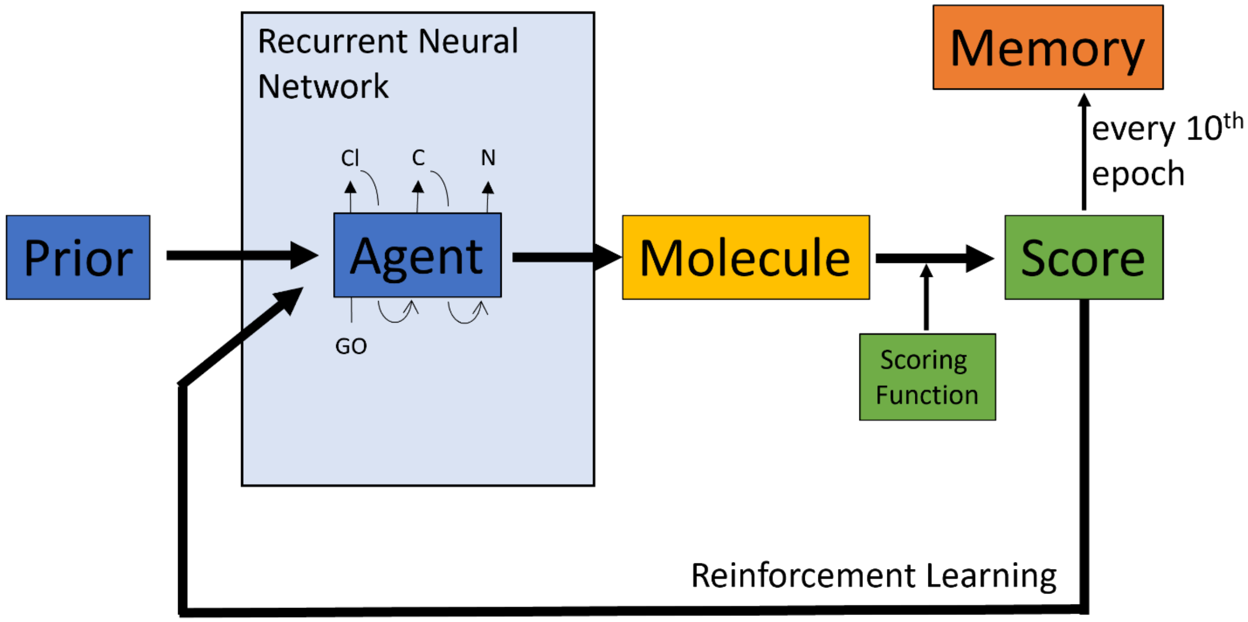

Supplementary Figure 1: Overview of the REINVENT training workflow.

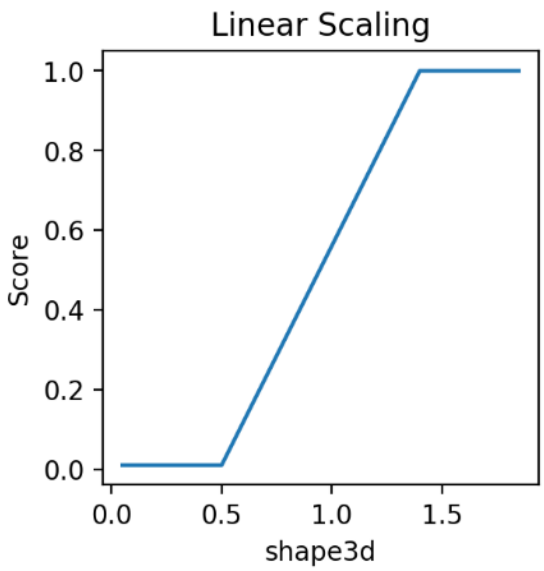

Supplementary Figure 2: Scaling of the ROCS-3d-similarity score. Lower threshold is 0.5, upper threshold is 1.4, everything in between is scaled linearly.

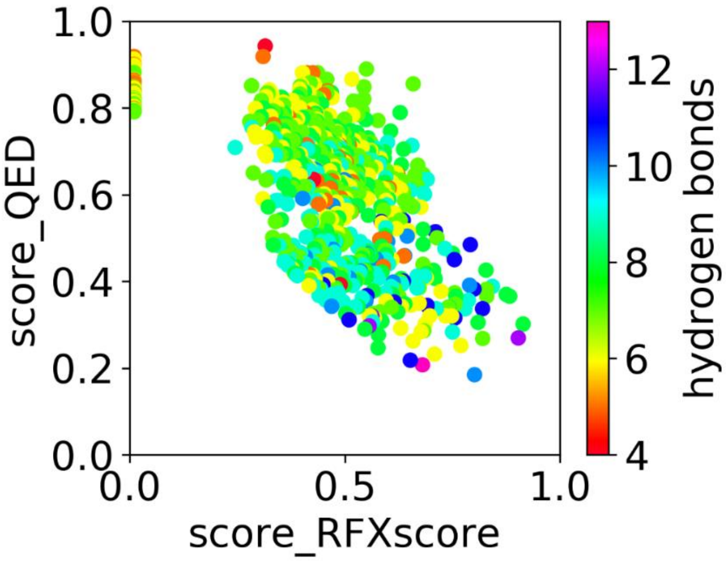

Supplementary Figure 3: Scatterplot of the scores QED and RFXscore for the sampled molecules from the RFXscore+QED run. The colors show the number of hydrogen bond donors and acceptors.

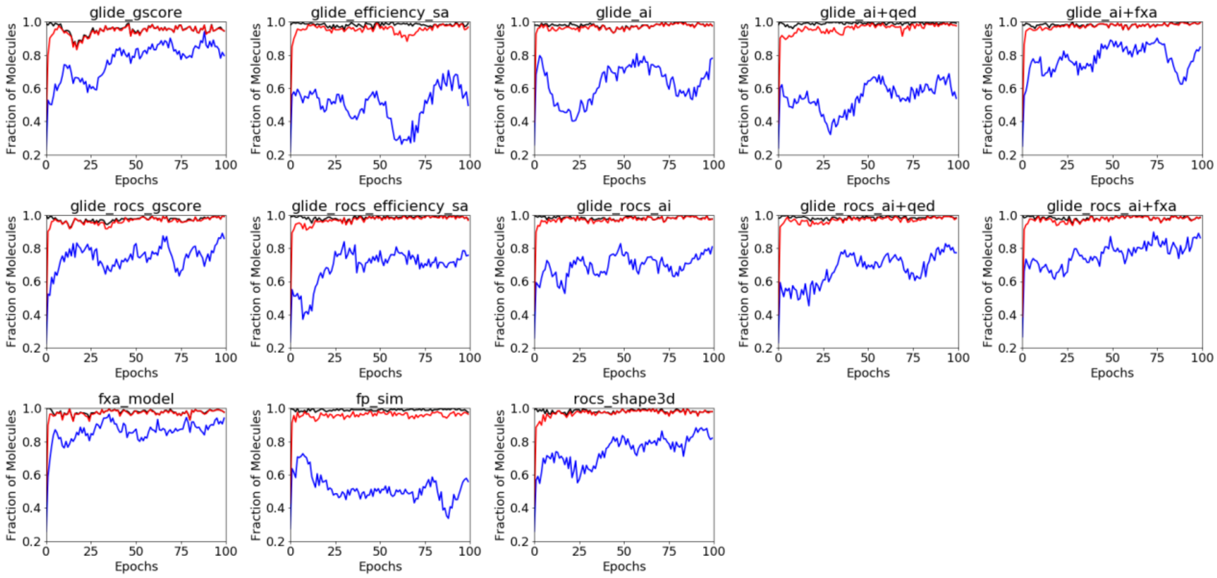

Supplementary Figure 4: Number of valid (black), unique (blue) and novel (red) molecules generated during Lib-INVENT training with different scoring functions

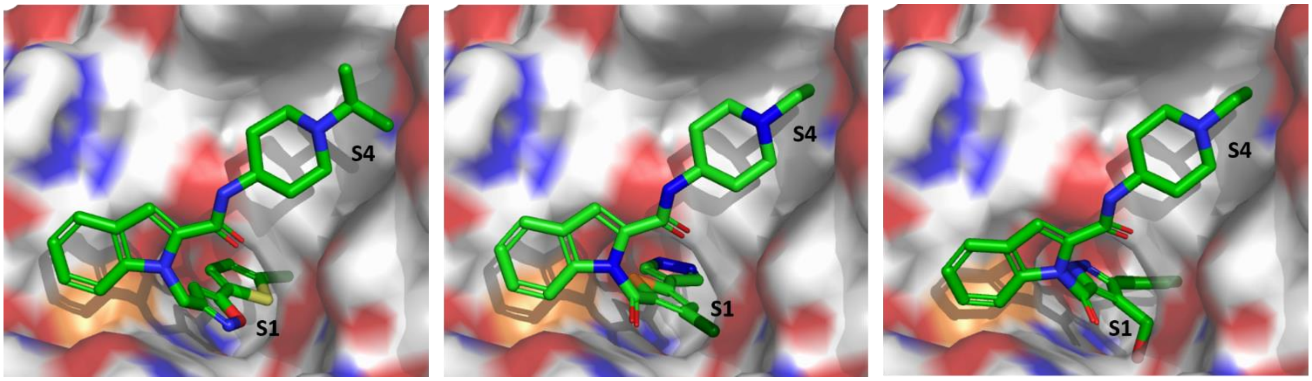

Supplementary Figure 5: Example of docked molecules generated with scoring function Glide-RFXscore+fXa (middle and right) in comparison to reference ligand (left). The atoms in the surface are colored by their element types.

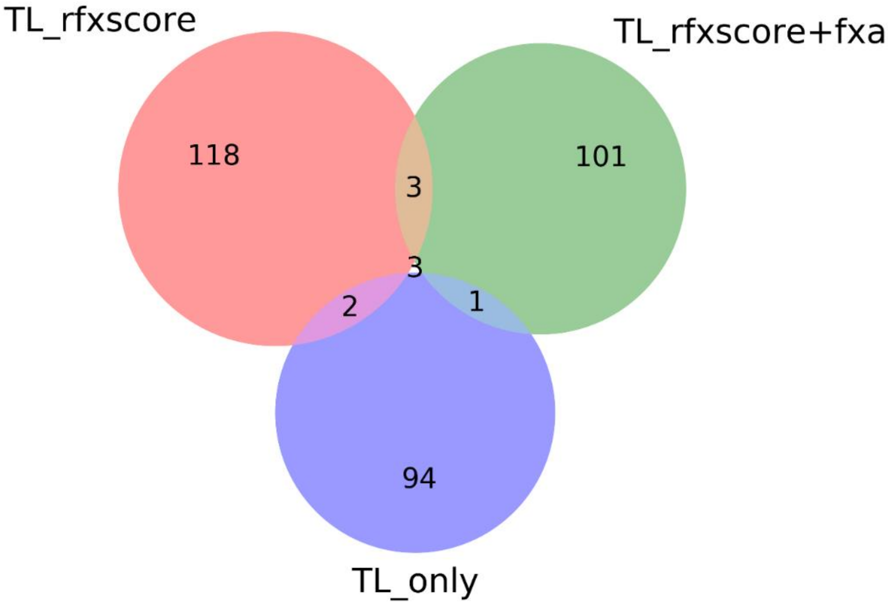

Supplementary Figure 6: Venn-Diagram of molecules after structural filters from the three runs using Transfer Learning

2 Supplementary Tables

Supplementary Table 1. Glide XP terms and description used to derive the scoring function.

See Schrödinger 2020-1 Release, Glide manual: XP Terms and their visualization for details.

- GlideXP_Score: Total GlideScore; sum of XP terms excluding Epik state penalties
- Glide_ligeff: Normalized GlideScore by number of heavy atoms.
- Glide_ligeff_sa: Normalized GlideScore by number of heavy atoms to the power of 2/3 to approximate the effect of the molecular surface area.
- Glide_ligeff_ln: Normalized GlideScore by 1+(ln(number of heavy atoms))
- Glide_rotatable_bonds: Penalty for freezing rotatable bonds.
- Glide_evdw: Van der Waals energy, calculated with reduced net ionic charges on groups with formal charges, such as metals, carboxylates, and guanidiniums.
- Glide_ecoul: Coulomb energy, calculated with reduced net ionic charges on groups with formal charges, such as metals, carboxylates, and guanidiniums.
- Glide_energy: Modified Coulomb-van der Waals interaction energy.
- Glide_einternal: Internal torsion energy.
- Glide_emodel: Model energy.
- Glide_XP_HBond: ChemScore H-bond pair term.
- Glide_XP_PhobEn: Reward for hydrophobic enclosure.
- Glide_XP_PhobEnHB: Reward for hydrophobically packed H-bonds.
- Glide_XP_LowMW: Reward for ligands with low molecular weight.
- Glide_XP_RotPenal: Rotatable bond penalty.
- Glide_XP_LipophilicEvdW: Lipophilic term derived from the hydrophobic grid potential at the hydrophobic ligand atoms.
- Glide_XP_PhobEnPairHB: Reward for hydrophobically packed correlated H-bonds.
- Glide_XP_Electro: Electrostatic rewards; includes Coulomb and metal terms.
- Glide_XP_SiteMap: SiteMap ligand-receptor non-H-bonding polar-hydrophobic and hydrophobic/hydrophilic complementarity terms
- Glide_XP_Penalties: Polar atom burial and desolvation penalties, and penalty for intra-ligand contacts. Those include: charge penalty, water-protein and water-ligand penalties, donor-donor penalty, penalty for intra-ligand contacts, penalty for burial of charged group on the protein by the ligand with no H bonds made to the charged group by the ligand or protein, penalty for desolvation of a polar ligand atom in a hydrophobic protein environment, penalty for putting a hydrophobic group of the ligand against polar (donor/acceptor) groups of the protein in a protein region which normally would give a favourable hydrophobic packing score.
- Glide_XP_PiStack: Reward for pi-stacking.
- Glide_XP_HBPenal: Penalty for ligands with large hydrophobic contacts and low H-bond scores.
- Glide_XP_ExposPenal: Penalty for solvent-exposed ligand groups; cancels van der Waals terms.

- Glide_XP_PiCat: Reward for pi-cation interactions.
- Glide_XP_ClBr: Reward for Cl or Br in a hydrophobic environment that pack against Asp or Glu.
- Glide_XP_Zpotr: Reward for ligand atoms in a favourable electrostatic environment of the protein.

Supplementary Table 2. RDKit ligand terms and description used to derive the scoring function.

See RDKit version 2021.09 documentation, https://www.rdkit.org/docs/GettingStartedInPython.html for details.

- NAtoms: Number of atoms
- NHev: Number of heavy atoms
- NHet: Number of hetero atoms:
- MolWt: Molecular weight
- logP: Computed logP
- TPSA: Topological polar surface area
- CSP3: Fraction of carbon C.sp3
- NOCount: N and O count
- NHOHCount: NH and OH count
- HAcc: Number of hydrogen bond acceptors
- HDon: Number of hydrogen bond donors
- NRot: Number of rotatable bonds
- NRings: Number of rings
- Naro: Number of aromatic rings
- Nsat: Number of saturated rings
- Nali: Number of aliphatic rings
- PEOE1-PEOE13: MOE-type subdivided surface area descriptors using partial charges and surface area contributions. See http://www.chemcomp.com/journal/vsadesc.htm
- SLOGP1-SLOGP12*: MOE-type subdivided surface area descriptors using LogP contributions and surface area contributions http://www.chemcomp.com/journal/vsadesc.htm
    - *Note: Values for SLOGP4 were not considered, as they consistently result in “nan” values.

Supplementary Table 3. Crossterms between Glide and RDKit used to derive the scoring function.

- GLXP1: GlideScore / Number of heavy atoms
- GLXP2: GlideScore / Number of hetero atoms
- GLXP3: GlideScore / logP

- GLXP4: GlideScore / TPSA
- GLXP5: GlideScore / Fraction of carbon C.sp3
- GLXP6: GlideScore / N and O count
- GLXP7: GlideScore / NH and OH count
- GLEVDW1: Glide Van der Waals energy / Number of heavy atoms
- GLEVDW2: Glide Van der Waals energy / Number of hetero atoms
- GLEVDW3: Glide Van der Waals energy / logP
- GLEVDW4: Glide Van der Waals energy / TPSA
- GLEVDW5: Glide Van der Waals energy / Fraction of carbon C.sp3
- GLEVDW6: Glide Van der Waals energy / N and O count
- GLEVDW7: Glide Van der Waals energy / NH and OH count
- GLECOUL1: Glide Coulomb energy / Number of heavy atoms
- GLECOUL2: Glide Coulomb energy / Number of hetero atoms
- GLECOUL3: Glide Coulomb energy / logP
- GLECOUL4: Glide Coulomb energy / TPSA
- GLECOUL5: Glide Coulomb energy / Fraction of carbon C.sp3
- GLECOUL6: Glide Coulomb energy / N and O count
- GLECOUL7: Glide Coulomb energy / NH and OH count
- GLE1: Glide modified Coulomb-van der Waals interaction energy / Number of heavy atoms
- GLE2: Glide modified Coulomb-van der Waals interaction energy / Number of hetero atoms
- GLE3: Glide modified Coulomb-van der Waals interaction energy / logP
- GLE4: Glide modified Coulomb-van der Waals interaction energy / TPSA
- GLE5: Glide modified Coulomb-van der Waals interaction energy / Fraction of carbon C.sp3
- GLE6: Glide modified Coulomb-van der Waals interaction energy / N and O count
- GLE7: Glide modified Coulomb-van der Waals interaction energy / NH and OH count
- GLHB1: Glide H-bond term / Number of heavy atoms
- GLHB2: Glide H-bond term / Number of hetero atoms
- GLHB3: Glide H-bond term / logP
- GLHB4: Glide H-bond term / TPSA
- GLHB5: Glide H-bond term / Fraction of carbon C.sp3
- GLHB6: Glide H-bond term / N and O count
- GLHB7: Glide H-bond term / NH and OH count
- GLLIPO1: Glide lipophilicity term / Number of heavy atoms
- GLLIPO2: Glide lipophilicity term / Number of hetero atoms
- GLLIPO3: Glide lipophilicity term / logP
- GLLIPO4: Glide lipophilicity term / TPSA
- GLLIPO5: Glide lipophilicity term / Fraction of carbon C.sp3
- GLLIPO6: Glide lipophilicity term / N and O count
- GLLIPO7: Glide lipophilicity term / NH and OH count

3 Outlier Discussion

The PDB file 1n4k with an affinity of 10.05, but a lower prediction of 5.90 is one of these outliers. This complex refers to the X-ray structure of the inositol 1,4,5-trisphosphate receptor binding core with IP3 at a resolution of 2.20 Å.(Bosanac et al., 2002) The ligand itself is a very small, polar cyclohexane derivative with three hydroxy-group and three charged phosphate residues, which are involved in strong salt bridges to the protein binding site. The prediction for this polar fragment suggests that multiple charge-based interactions of smaller fragments are not captured correctly.

The PDB file 7std is also mispredicted with an experimental affinity of 10.72, but a predicted value of 6.998, respectively. This corresponds to the X-ray structure of scytalone dehydrase with its inhibitor at a resolution of 1.80 Å.(Wawrzak et al., 1999) The inhibitor is a very lipophilic, fragment-like molecule with only one weak, polar interaction and a single halogen-bond interaction involving a chlorine and a neighboring backbone amide proton. However, the majority of interactions is lipophilic. Interestingly, this compound is reported in the literature with a surprisingly high picomolar binding affinity, which is not fully reflected in the predictions.

The PDB file 3s73 provides another example of a mispredicted fragment with a significant polar interaction to the binding site. This complex refers to the X-ray structure of carbonic anhydrase 2 (CA-II) with a small arylsulfonamide at a resolution of 1.75 Å with high binding affinity.(Snyder et al., 2011) While an experimental affinity of 8.70 was observed, only a value of 5.13 is predicted. A zinc-ion is present in the active site of CA-II, for which key interactions to the polar head group are observed. Furthermore, interactions for the aromatic, small fragment with high potency are present, although not predicted correctly.

The PDB file 3zt3 provides an example of a complex with very weak experimental affinity (2.86), while a significantly higher value of 6.328 was predicted. The corresponding fragment-based inhibitor interacts with the LEDGF site of HIV type 1 integrase. It was identified by fragment screening and solved at a resolution of 1.95 Å.(Peat et al., 2012) This fragment exhibits a surprisingly low biological activity, while its X-ray structure reveals many favorable polar interactions of its carboxylate headgroup plus additional lipophilic interactions to the protein binding site. It should be noted that the experimental data are based on biophysical SPR measurements, which might also add uncertainty in this low affinity range.

The PDB file 1kuk refers to X-ray structure of an exotic metalloproteinase with an inhibitor at a very high resolution of 1.45 Å.(Huang et al., 2002) While a surprisingly low biological activity was reported from the experimental assays (3.91), a significantly higher value of 7.733 was predicted. The inhibitor is a tripeptide showing good and complementary interactions to the protein binding site. One important feature is the interaction of a carboxylate to the binding site metal (here Cadmium).

Finally, the PDB file 5cs3 refers to the complex structure of the NK1 fragment of HGF/SF complexed with (H)EPPS, solved at a resolution of 2.5 Å.(Sigurdardottir et al., 2015) While its experimental activity is very low from SPR measurements with a value of 2.15, a higher value of 6.292 is predicted. The structure is a very small and highly polar, charged molecule exhibiting salt bridges and cation-π interactions to the protein binding site. There is no obvious structural reason for the observed low activity of this fragment.

In summary, these examples suggest that affinities for smaller fragments with very polar or lipophilic interactions to binding sites are more difficult to capture. Our scoring function was not trained in

particular for smaller fragments (Wang and Lin, 2015), while it aimed for describing leads and drug-like structures sufficiently. Some examples also highlight the importance to employ consistent biological data from high quality assays.(Sotriffer et al., 2008) However, in practice it is often difficult to obtain a chemically and biologically diverse dataset like PDBbind for development of relevant scoring functions based on only high-quality biological data.(Volkov et al., 2022) This finding prompted us to enrich the function with ligand SAR information from relevant projects.

Supplementary Table 4. List of outliers in testset for RFXscore model

PDB	Exp	Pred	Residual
1n4k	10.05	5.906	4.144
5sz2	8.70	4.678	4.022
7std	10.72	6.998	3.722
3s73	8.70	5.130	3.570
1d4y	11.10	7.781	3.319
1b8n	10.52	7.250	3.270
5eei	9.22	5.973	3.247
3dd0	9.00	5.779	3.221
1g2o	10.55	7.381	3.169
6hke	7.68	4.513	3.167
1n46	10.52	7.417	3.103
4m8x	10.20	7.159	3.041
2std	9.85	6.834	3.016
--			
3t08	3.04	6.081	-3.041
4rrg	2.81	5.886	-3.076
3ozt	4.13	7.297	-3.167
1f73	2.39	5.669	-3.279
5azf	4.44	7.721	-3.281
3ao5	2.23	5.561	-3.331
4ahu	2.27	5.616	-3.346
4ahs	2.19	5.586	-3.396
1m0o	2.31	5.743	-3.433
3zt3	2.86	6.328	-3.468
3iae	3.89	7.49	-3.600
1kuk	3.91	7.733	-3.823
5cs3	2.15	6.292	-4.142
4qgi	3.90	8.26	-4.360

4 Literature

BOSANAC, I., ALATTIA, J.-R., MAL, T. K., CHAN, J., TALARICO, S., TONG, F. K., TONG, K. I., YOSHIKAWA, F., FURUICHI, T., IWAI, M., MICHIKAWA, T., MIKOSHIBA, K. & IKURA, M. 2002. Structure of the inositol 1,4,5-trisphosphate receptor binding core in complex with its ligand. *Nature,* 420**,** 696-700.

HUANG, K.-F., CHIOU, S.-H., KO, T.-P. & WANG, A. H.-J. 2002. Determinants of the inhibition of a Taiwan habu venom metalloproteinase by its endogenous inhibitors revealed by X-ray crystallography and synthetic inhibitor analogues. *European Journal of Biochemistry,* 269**,** 3047-3056.

PEAT, T. S., RHODES, D. I., VANDEGRAAFF, N., LE, G., SMITH, J. A., CLARK, L. J., JONES, E. D., COATES, J. A. V., THIENTHONG, N., NEWMAN, J., DOLEZAL, O., MULDER, R., RYAN, J. H., SAVAGE, G. P., FRANCIS, C. L. & DEADMAN, J. J. 2012. Small molecule inhibitors of the LEDGF site of human immunodeficiency virus integrase identified by fragment screening and structure based design. *PloS One,* 7**,** e40147.

SIGURDARDOTTIR, A. G., WINTER, A., SOBKOWICZ, A., FRAGAI, M., CHIRGADZE, D., ASCHER, D. B., BLUNDELL, T. L. & GHERARDI, E. 2015. Exploring the chemical space of the lysine-binding pocket of the first kringle domain of hepatocyte growth factor/scatter factor (HGF/SF) yields a new class of inhibitors of HGF/SF-MET binding. *Chemical Science,* 6**,** 6147-6157.

SNYDER, P. W., MECINOVIC, J., MOUSTAKAS, D. T., THOMAS, S. W., HARDER, M., MACK, E. T., LOCKETT, M. R., HEROUX, A., SHERMAN, W. & WHITESIDES, G. M. 2011. Mechanism of the Hydrophobic Effect in the Biomolecular Recognition of Arylsulfonamides by Carbonic Anhydrase. *Proc. Natl. Acad. Sci. USA,* 108**,** 17889-17894.

SOTRIFFER, C. A., SANSCHAGRIN, P., MATTER, H. & KLEBE, G. 2008. SFCscore: Scoring functions for affinity prediction of protein–ligand complexes. *Proteins: Structure, Function, and Bioinformatics,* 73**,** 395-419.

VOLKOV, M., TURK, J.-A., DRIZARD, N., MARTIN, N., HOFFMANN, B., GASTON-MATHÉ, Y. & ROGNAN, D. 2022. On the Frustration to Predict Binding Affinities from Protein-Ligand Structures with Deep Neural Networks. *Journal of Medicinal Chemistry,* 65**,** 7946-7958.

WANG, J.-C. & LIN, J.-H. 2015. Scoring functions for fragment-based drug discovery. *Methods in Molecular Biology (Clifton, N.J.),* 1289**,** 101-115.

WAWRZAK, Z., SANDALOVA, T., STEFFENS, J. J., BASARAB, G. S., LUNDQVIST, T., LINDQVIST, Y. & JORDAN, D. B. 1999. High-resolution structures of scytalone dehydratase-inhibitor complexes crystallized at physiological pH. *Proteins,* 35**,** 425-439.
